# Femtosecond Laser Fabrication of Anisotropic Structures in Phosphorus- and Boron-Doped Amorphous Silicon Films

**DOI:** 10.3390/ma15217612

**Published:** 2022-10-29

**Authors:** Dmitrii Shuleiko, Stanislav Zabotnov, Mikhail Martyshov, Dmitrii Amasev, Denis Presnov, Vyacheslav Nesterov, Leonid Golovan, Pavel Kashkarov

**Affiliations:** 1 Faculty of Physics, Lomonosov Moscow State University, 1/2 Leninskie Gory, 119991 Moscow, Russia; 2 Prokhorov General Physics Institute of the Russian Academy of Sciences, 38 Vavilova st., 119991 Moscow, Russia; 3 Skobeltsyn Institute of Nuclear Physics, Lomonosov Moscow State University, 1/2 Leninskie Gory, 119991 Moscow, Russia; 4 Quantum Technology Centre, Lomonosov Moscow State University, 1/35 Leninskie Gory, 119991 Moscow, Russia; 5 National Research Centre “Kurchatov Institute”, 1 Akademika Kurchatova sq., 123182 Moscow, Russia

**Keywords:** femtosecond laser pulses, laser-induced periodic surface structures, surface plasmon-polaritons, amorphous silicon, Raman spectroscopy, electrophysical measurements

## Abstract

Femtosecond laser-modified amorphous silicon (a-Si) films with optical and electrical anisotropy have perspective polarization-sensitive applications in optics, photovoltaics, and sensors. We demonstrate the formation of one-dimensional femtosecond laser-induced periodic surface structures (LIPSS) on the surface of phosphorus- (n-a-Si) and boron-doped (p-a-Si) amorphous silicon films. The LIPSS are orthogonal to the laser polarization, and their period decreases from 1.1 ± 0.1 µm to 0.84 ± 0.07 µm for p-a-Si and from 1.06 ± 0.03 to 0.98 ± 0.01 for n-a-Si when the number of laser pulses per unit area increases from 30 to 120. Raman spectra analysis indicates nonuniform nanocrystallization of the irradiated films, with the nanocrystalline Si phase volume fraction decreasing with depth from ~80 to ~40% for p-a-Si and from ~20 to ~10% for n-a-Si. LIPSS’ depolarizing effect, excessive ablation of the film between LIPSS ridges, as well as anisotropic crystalline phase distribution within the film lead to the emergence of conductivity anisotropy of up to 1 order for irradiated films. Current–voltage characteristic nonlinearity observed for modified p-a-Si samples may be associated with the presence of both the crystalline and amorphous phases, resulting in the formation of potential barriers for the in-plane carrier transport and Schottky barriers at the electric contacts.

## 1. Introduction

Direct laser writing (DLW) is an effective approach to structure solid states with micron and submicron accuracy for microelectronics and photonics. In comparison to lithographic techniques, DLW usually needs only irradiation and does not demand such additional stages as template preparation and chemical etching. Laser-induced periodic surface structures (LIPSS) are bright examples of applying the DLW technique for structuring of metals, semiconductors and dielectrics. It is possible to fabricate different types of LIPSS: low-spatial-frequency LIPSS (LSFL) are gratings with the spatial period close to the wavelength of the excitation pulse [1,2,3,4,5]; high-spatial-frequency LIPSS (HSFL) [6,7,8,9,10,11] and supra-wavelength (SWPSS) [12,13] are periodic surface structures where the period is significantly smaller or larger than the wavelength of the incident light, respectively, as well as circular-LIPSS (CLIPPS) [14,15,16], and are hierarchical structures which are superpositions of the listed types of LIPSS [17,18,19,20].

Traditionally, two main classes of theories for LIPSS formation are applied [21,22]. (i) One is an electromagnetic approach, where LIPPS appear due to interference of photoexcited surface plasmon-polaritons (SPP) and incident laser pulses. Such theories allow us to explain not only LSFL fabrication [1,2,3,4,5,23,24] but also HSFL occurrence, with consideration of local field redistribution [9,25,26] or second-harmonic generation [10,11] in a near-surface layer. Usually, this approach is applicable for the description of ultrafast photoexcitation of surface by laser pulses with a duration of not more than several picoseconds. (ii) Matter reorganization theories which demand to take into account self-organization hydrodynamic processes in a melt meaning Marangoni convection, capillary or thermoelastically acoustic waves [7,12,13,16,22]. Such mechanisms usually manifest themselves at the times that exceed the laser pulse duration and allow us to explain fabrication a wide variety of LIPSS with the different periods and morphologies. It is necessary to mention that both approaches do not exclude each other and may give comparable contribution into LIPPS fabrication [13,17,27].

From a practical point of view, LIPPS’ existence may lead to a noticeable artificial anisotropy of optical and electrophysical properties of irradiated surfaces [3,4,28,29]. A specific application of such anisotropic structures is defined by properties and features of the irradiated matter. In the last decade, considerable interest has been shown to femtosecond laser irradiation of thin amorphous silicon (a-Si) films. Such treatment generates not only the occurrence of LIPPS [3,4,18,28], but also suppression of photodegradation and the rise of both optical absorption [30,31] and electrical conductivity in the irradiated a-Si film [4,32]. Therefore, the femtosecond laser processing of a-Si opens new prospects for photovoltaics. Herein it is important that this technology is a complex process that affects not only the surface of the material, but also its phase composition, e.g., formation of a significant crystalline Si (c-Si) volume fraction in the a-Si film [28,32,33]. As a result, modulation of the crystalline and amorphous phases distribution can occur within the formed LIPSS [3,18], along with the relief modulation affect the anisotropy of the optical and electrical properties of a-Si film, which may be applicable for polarization-sensitive optics and sensors [18,28]. Another promising application of the periodically modified a-Si films are all-dielectric optical metasurfaces [34] and phase-change gratings [35].

Nevertheless, current experiments on femtosecond LIPPS fabrication were carried out with undoped a-Si films [3,4,18,28,30,34], whereas laser irradiation of doped a-Si films and consequent LIPSS formation should cause strong electrical anisotropy, which is of great interest for photovoltaic and polarization-sensitive applications. However, LIPSS formation on the surface of doped a-Si films has not been studied yet [21]. Therefore, in our work we set the aim to fabricate LIPSS on the a-Si films doped with phosphorus or boron at a different number of irradiating laser pulses, and analyze the effect of the formed surface relief and phase transitions on the electrical properties of these films.

## 2. Materials and Methods

**Figure 1:**
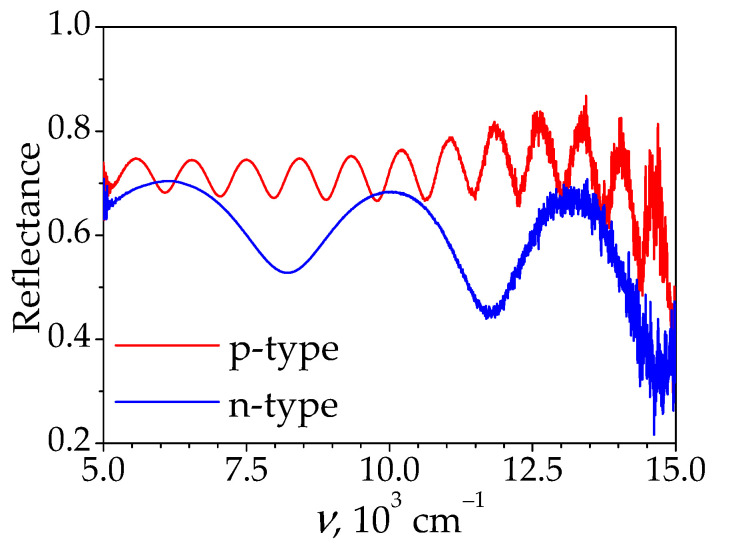
Reflectance spectra of unmodified a-Si films.

Initial boron-doped p-type a-Si films (p-a-Si) and phosphorus-doped n-type a-Si films (n-a-Si) were fabricated by plasma-enhanced chemical vapor deposition on glass substrates. The thickness of the films was determined from the analysis of IR reflection spectra (Figure 1), obtained using a Bruker IFS-66v/S (Bruker Optics, Ettlingen, Germany) infrared Fourier spectrometer at a 13° angle of incidence in the near-infrared diapason, and is given in Table 1.

**Figure 2:**
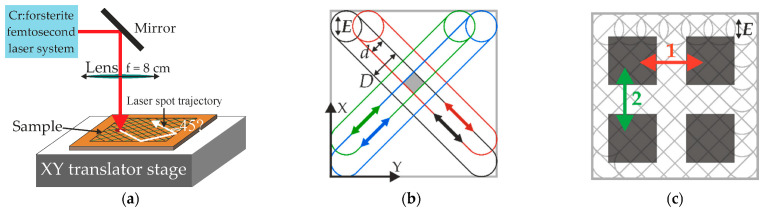
(**a**) Scheme of experimental setup used to process the a-Si films by femtosecond laser pulses. (**b**) Scanning mode template of femtosecond laser irradiation. Four different laser beam passes are indicated by black, red, green, and blue colors. The arrow *E* indicates laser polarization. (**c**) Scheme of the electrical contacts (gray squares) on the irradiated sample.

Fabricated films were irradiated by an Avesta pulsed femtosecond laser system (Avesta-Project, Moscow, Russia) with the wavelength *λ* = 1250 nm, pulse duration *τ* = 150 fs, and repetition rate *f* = 10 Hz. The irradiation was carried out at a normal incidence using a laser spot with a diameter of *D* = 150 μm. The sample irradiation in the scanning mode was realized by moving the film relative to the laser beam on the system of automated translators (Standa Ltd., Vilnius, Latvia) along two mutually orthogonal axes, X and Y, in the horizontal plane. The scheme of the setup is given in Figure 2a.

The film was moved smoothly and simultaneously along both axes at the same speed, *V*. As a result, the scan lines were formed diagonally on the processed region at an angle of 45° to its edge, while the polarization of laser pulses was directed along the X axis in all cases. Such a scanning regime will make a dominant contribution to in-plane anisotropy of the exact LIPSS at a minimal influence of so-called scan lines, which are tracks from the laser beam on the irradiated surface. For comparison, the often-used meander-like scanning strategy may give the dominant contribution of the scan lines to the electrophysical anisotropy, as was shown in our previous work [29].

To evaluate the effect of the number of laser pulses on the irradiation result, 2 different scanning speeds were used (Table 1), while other processing parameters (pulse fluence and duration, wavelength, angle of incidence) were fixed. Thus, on the surface of both the p-a-Si and n-a-Si films, 2 samples in the form of square 5 × 5 mm regions were formed. For more uniform processing, the lateral overlap of the scan lines *d* was two times smaller than the diameter of the laser spot *D.* It should be noted that further reducing the lateral overlap is undesirable, as it will lead to increase in the laser beam passes number, which may lead to a disordering of the formed surface periodic structure due to the deposition of ablation products after each pass.

The scanning of each region was performed twice, along both diagonals of the scanned square. As a result, each point of the sample was passed by the laser beam 4 times, as demonstrated on the scheme in Figure 2b, where each of the 4 passes is designated by a different color.

Consequently, the total irradiation dose was determined by the number *N* of laser pulses acting on the unit area of the sample:

*N* = 4∙*fD*/*V*. (1)

**Figure 3:**
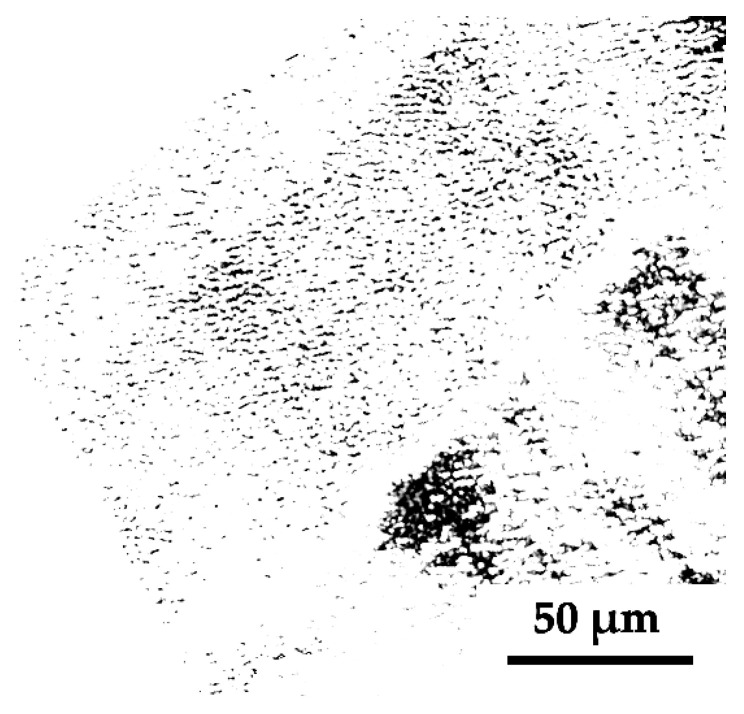
Image of the p-a-Si surface irradiated with *N* = 200, obtained by optical microscope in transmission mode. Dark sites are silicon isles surround surrounded by empty space, arisen because of complete film ablation.

The fluences of the laser radiation used for processing the films, as well as other sample processing parameters are given in Table 1. The choice of a higher fluence in case of p-a-Si film is explained by its greater thickness (Supplementary file S1, [36,37]). We should also note that for the samples of both doping types, irradiation by more than 200 pulses completely removes silicon in the LIPSS valleys, leaving isolated silicon isles at the glass substrate (Figure 3).

Images of the irradiated samples on the surface of the films were obtained by a scanning electron microscope (SEM), a Carl Zeiss Supra 40 (Carl Zeiss AG, Oberkochen, Germany) and Olympus BX41 (Olympus Corporation, Tokyo, Japan) optical microscope. The depth of the surface relief was estimated from the SEM images of the cross-section of the irradiated regions. The period of the surface relief was obtained from the SEM images analysis. For each sample, 10 measurements of the length of 10 LIPSS periods were carried out, and then the obtained values were averaged for each sample.

The Raman spectra used for the composition analysis of both initial and modified a-Si films were obtained using Horiba HR 800 Raman microscope (Horiba Jobin Yvon GmbH, Bensheim, Germany) at 488 nm excitation. The measurements were carried out both at the front side of the films and at their back side, through the glass substrate.

To analyze the electrical properties of the initial and modified a-Si films, the aluminum contacts in groups of 4 (Figure 2c) were deposited on the surface of each irradiated region, as well as on untreated areas, by thermoresistive sputtering. On the modified regions the contact sides were directed parallel to their boundaries, i.e., at a 45° angle to the scan lines direction. This provided the possibility to carry out measurements in the surface plane in two mutually orthogonal directions and analyze the electrical anisotropy of the samples induced by LIPSS, while the effect of the formed scan lines was negated. A Keithly 6487 picoammeter (Keithley Instruments Inc., Singapore) was used to measure the dark conductivity of the samples.

## 3. Results

### 3.1. Surface Periodic Relief Period and Depth

**Figure 4:**
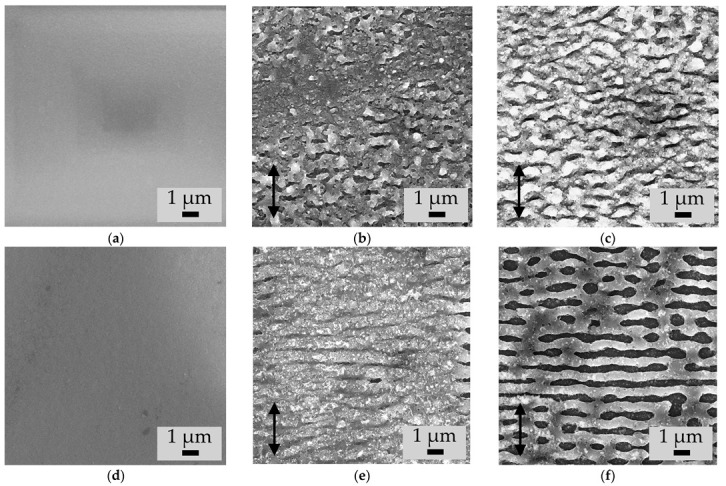
SEM images of the surfaces of (**a**) initial p-a-Si; femtosecond laser-irradiated samples (**b**) P1, (**c**) P2; (**d**) initial n-a-Si; femtosecond laser-irradiated samples (**e**) N1 and (**f**) N2. The direction of laser polarization is indicated by an arrow on the images.

The LIPSS formation was revealed by SEM on the surface of the irradiated a-Si films with both doping types (Figure 4). The period of these structures in all cases was shorter than the wavelength of the laser radiation used, but close to it (see Table 1). In all cases the LIPSS ridges are directed orthogonally to the laser radiation polarization. The structures on the p-a-Si films surface are weakly ordered, compared to more distinct one-dimensional gratings on the n-a-Si films (cf. Figure 4b,c,e,f), which is possibly caused by thermal processes in thicker p-a-Si film. The initial surface in cases of both p-a-Si and n-a-Si films is smooth, as demonstrated in Figure 4a,d.

**Figure 5:**
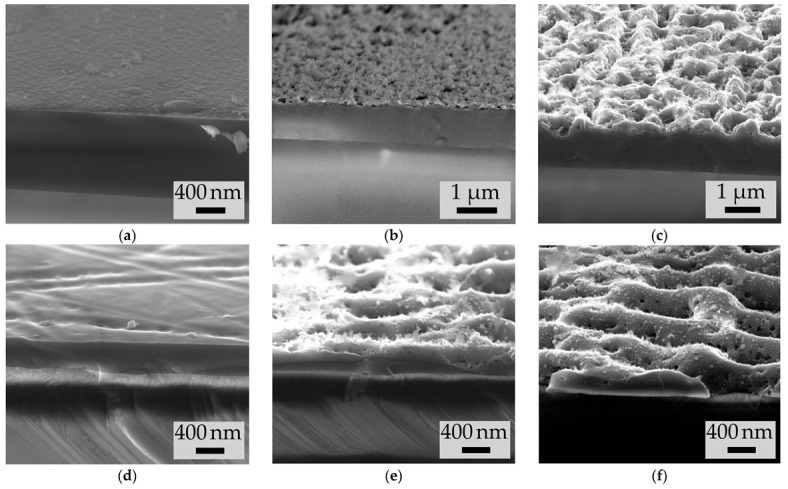
SEM images of the cross sections of the samples (**a**) unirradiated p-a-Si (**b**) P1, (**c**) P2, (**d**) unirradiated n-a-Si (**e**) N1 and (**f**) N2, obtained at a 10° angle to the cross section surface.

For the both types of a-Si films the LIPSS period decreased as *N* increased from 30 to 120 (Table 1). Additionally, with increasing *N*, the depth of the formed relief also increased, as can be seen in the SEM images of the cross sections of the films (Figure 5). In the case of p-a-Si film, the relief depth increased from ~100 nm for the P1 region and to ~300 nm for the P2 region. The relief on the n-a-Si film was almost 2 times deeper, despite the lower fluence used: the depth increased from ~200 nm for the N1 region, which is about the half of the film thickness, to the entire thickness of the n-a-Si film (~350 nm). Thus, for the N2 sample, complete ablation of the n-a-Si film was observed in the valley between LIPSS ridges (Figure 4f and Figure 5f). More intense ablation of the n-a-Si film may be explained by the smaller thickness of this film and, as a result, a less efficient heat removal.

### 3.2. Raman Spectra Analysis

**Figure 6:**
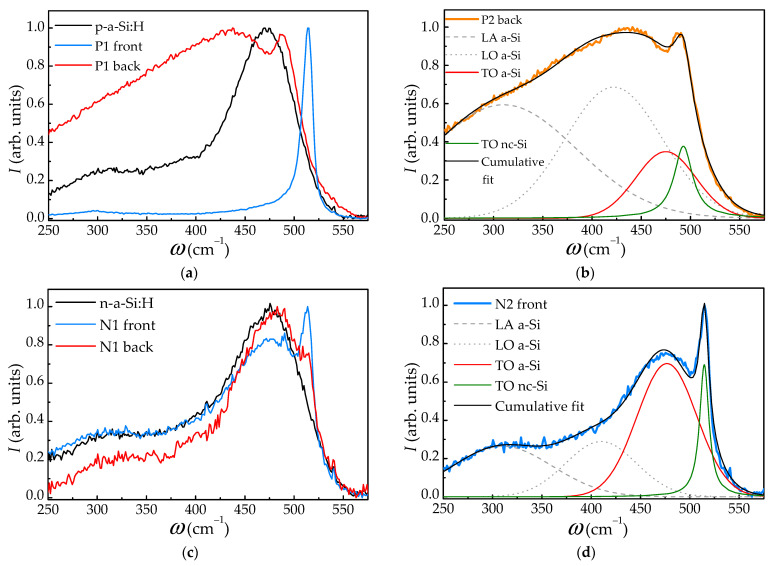
(**a**) Typical Raman spectra for the initial p-a-Si film and irradiated region P1. (**b**) Decomposition of the Raman spectrum obtained from the back of the sample P2. (**c**) Typical Raman spectra for the initial n-a-Si film and irradiated region N1. (**d**) Decomposition of the Raman spectrum for the front of the sample N2. All spectra are normalized by maximum intensity.

The typical Raman spectra of irradiated regions, as well as unmodified films are given in Figure 6a,c. Each spectrum was normalized by a maximum spectrum intensity. To analyze the obtained spectra, we carried out their decomposition into bands corresponding to amorphous and crystalline phases (Figure 6b,d). Raman spectra of both unmodified p-a-Si and n-a-Si films demonstrate bands corresponding to LA, LO, and TO a-Si phases centered at 310, 420, 480 cm^−1^, respectively [38,39]. The spectra of irradiated regions indicate formation of a crystalline (nanocrystalline) silicon phase (nc-Si) in the p-a-Si and n-a-Si films as a result of femtosecond laser irradiation, which manifests itself in the presence of a corresponding narrow TO nc-Si line near *ω*_C_ = 520 cm^−1^. Note the difference in the Raman spectra obtained at the front and back sides of the irradiated regions (Figure 6a,c), indicating inhomogeneous crystallization of both types of the films. For example, in the Raman spectrum of the modified p-a-Si film front side, a line corresponding to ω_C_ is observed, albeit at lower a Stokes shift (518 cm^−1^), while the band near ω_A_ = 480 cm^−1^ is not visible. However, for the reverse side of the film, the shift of the ω_C_ line towards lower Stokes shift values is higher (Figure 6a,b). These observations indicate the formation of Si nanocrystals in the film as a result of laser irradiation. The Raman line shifts are caused by the quantum size effect for phonons of this mode [40]. The determined nanocrystals sizes *d_nc-Si_*, for both the frontal side and back of modified p-a-Si and n-a-Si films, were calculated according to Ref. [40] and are given in Table 2. In the case of n-a-Si films, the intensity of the ω_C_ line decreases on the reverse side of the film, but its position does not change.

The nanocrystalline phase volume fraction *f_C_* in the irradiated films can be calculated from the Raman spectra using the expression [4,41]:

(2) $$f_{C}=\frac{I_{C}}{\sigma_{0}I_{A}+I_{C}},$$

where *I_A_* and *I_C_* are the integral intensities of the TO phonon modes corresponding to lines near the frequencies *ω_A_* and *ω_C_* in the Raman spectrum, and *σ*_0_ is the empirical ratio of the Raman-scattering integral cross sections for the nc-Si and a-Si phases, which is determined by the size of the formed silicon nanocrystals according to the formula [4,42]:

*σ*_0_ = 0.1 + exp(−*d_nc-Si_*/25). (3)

The calculated *f_C_* values are given in Table 2.

### 3.3. Electrical Properties

**Figure 7:**
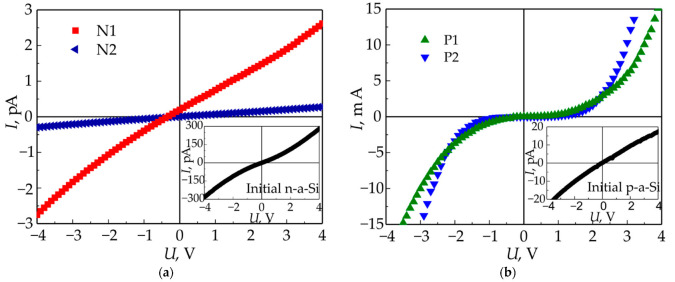
(**a**) CVC of irradiated p-a-Si films. (**b**) CVC of irradiated n-a-Si films. The insets show the CVC of the initial p-a-Si and n-a-Si films, respectively.

For all irradiated areas, the current–voltage characteristics (CVC) were measured in the surface plane, in two mutually orthogonal directions. In this case, the electric field was applied either along or orthogonally to the LIPSS, and the effect of scanning direction on the electrophysical properties was minimized due to the scan lines formation at an angle of 45° to the edges of the treated areas. The CVC characteristics of irradiated areas are shown in Figure 7, while the CVC characteristics of the initial p-a-Si and n-a-Si are given in the insets in corresponding figures.

As can be seen in Figure 7a, the CVC obtained for the initial p-a-Si film and shown in the inset is linear, while the CVC characteristics of both irradiated regions P1 and P2 on the surface of p-a-Si are non-linear. At the same time, the CVC of both initial and irradiated n-a-Si is almost linear (Figure 7b).

The specific conductivities for both initial and irradiated regions of p-a-Si and n-a-Si films calculated from the CVC at U = 1 V are given in Table 3. Hereinafter, the CVC characteristics of irradiated samples measured along the LIPSS have the “1” postscript, and CVC measured orthogonally to the LIPSS have the “2” postscript (Figure 2c). As can be seen from Table 3, the specific conductivity of the p-a-Si film increased significantly (by 6–7 orders of magnitude) after the exposure to high-power femtosecond laser pulses, while for the n-a-Si film the specific conductivity increase is not so significant, and in the case of sample area N2–2 a decrease in specific conductivity was observed.

In addition, the conductivity of all irradiated samples demonstrates significant anisotropy: when the electric field ***E*** is applied along the LIPSS, its value is from ~3.5 to ~13 times more than in the case when ***E*** is directed orthogonally to the LIPSS (Table 3).

## 4. Discussion

### 4.1. Surface Periodic Structures Formation and Nanocrystallization of Irradiated Films

The formation of LIPSS by femtosecond laser pulses, with ridges directed orthogonally to laser polarization, is associated with the generation of SPP on the silicon surfaces due to the intense photoexcitation of a nonequilibrium electron-hole plasma there under the action of high-power laser radiation. Such photoexcitation leads to metallization of the near-surface region during the irradiation when surface dielectric permittivity dramatic reduces and becomes a negative value in the irradiated region according to the Drude model [1,22,43]. The period of the formed LIPSS is determined by the laser radiation wavelength, laser beam angle of incidence, and concentration of nonequilibrium electrons excited by laser pulses [2,22,44].

The observed decrease in the surface gratings period with an increase in the number of irradiating laser pulses, both in the case of p-a-Si and n-a-Si, can also be explained within the framework of the plasmon-polariton theory of LIPSS formation. As shown above, the depth of the LIPSS increases with the number of acting laser pulses *N*, which according to Ref. [24], shifts the SPP resonant period towards lower values with the result that LIPSS with a lower period are formed. On the other hand, when the relief depth increases, the absorption coefficient of the irradiated structure also increases [24]. This, in its turn, increases the absorbed laser radiation energy. Therefore, the excited nonequilibrium charge carriers concentration, and their temperature also increases, which leads to an increase in the modulus of the dielectric permittivity *ε* real part of the material. This should lead to an increased period of the formed LIPSS, bringing its value closer to the acting laser radiation wavelength λ according to the formula for the considered case of the normal laser beam incidence [22]:

(4) $$\Lambda =\lambda \sqrt{\frac{\left|Re\left(\varepsilon \right)\right|-1}{\left|Re\left(\varepsilon \right)\right|}},$$

However, such more intense heating of the electronic subsystem of a film by femtosecond laser pulses [45] can also lead to an increase in the thermal emission of electrons from the material surface [43,46]. This, as a result, leads to a decrease in concentration of nonequilibrium charge carriers in the film, and, consequently, a decrease in the LIPSS period too. As a result, the observed LIPSS behavior allows their classification as LSFL.

Note that in both the cases of p-a-Si and n-a-Si films, no rotation of the LIPSS was observed up to the *N* = 200; conversely, in the case of an undoped a-Si film for the same *N* the formation of structures directed along the laser polarization vector was observed in our previous experiments [3].

In addition to the LIPSS formation, the femtosecond laser irradiation led to nonuniform nanocrystallization of the films. The value of *f_C_* decreases with depth by ~2 times, from ~80% on the film surface to ~40% near the substrate for p-a-Si and from ~20% to ~10%—for n-a-Si, as can be seen from Table 2. This effect is associated with a decrease in the intensity of transmitted laser radiation due to its high absorption, both in the single-photon and double-photon regimes, by relatively thick a-Si films. However, *f_C_* does not significantly change when the N is increased from 30 to 120. Such accumulation of nc-Si phase does not occur, most likely, due to the fast and well-pronounced ablation of the near-surface layer evidenced by the formation of a deeper surface relief (Figure 5).

### 4.2. Influence of the Structural Changes on the Electrical Properties of Irradiated Films

The increase in p-a-Si film-specific conductivity by 6–7 orders of magnitude after femtosecond laser irradiation is explained by the formation of the nc-Si phase with high volume fraction *f_C_* up to ~80%, as was shown by Raman spectra analysis. It is important to note that the modification of samples P1 and P2 with different numbers of laser pulses did not lead to a significant difference in their specific conductivities, which also agrees with close *f_c_* values for these samples obtained from Raman spectra analysis.

A less pronounced increase in specific conductivity of n-a-Si film can be explained by weak crystallization, which is characterized by *f_c_* < 20% (Table 2), as well as a highly specific conductivity of the initial n-a-Si film: its value is almost 3 orders of magnitude higher than the conductivity of the original p-a-Si film. High conductivity of untreated n-a-Si is associated with a much higher electron drift mobility in it *μ*_De_ = 0.1 cm^2^/(V∙s), compared to the hole mobility in p-a-Si *μ*_Dh_ = 5 × 10^−4^ cm^2^/(V∙s) [47,48]. The concentrations of the main charge carriers *n_e,h_* in the films—electrons in n-a-Si and holes in p-a-Si—are close in magnitude: *n_e_* ≈ 10^14^ cm^−3^ and *n_h_* ≈ 3 × 10^13^ cm^−3^, estimated according to the formulae

*n_e_* = *σ_n,_/eμ_De_*, *n_h_* = *σ_p_/eμ_Dh_*, (5)

where *σ_n,p_* is the specific conductivity of the initial n- and p-type films from Table 3, respectively, and *e* is the elementary electric charge. Undoped a-Si has weak properties of an n-type semiconductor [49,50], whereas p-type doping converts it to compensated semiconductor. Crystallization caused by the laser treatment activates both p- and n-type impurities [51]. Besides, the crystallization results in disappear of the compensation, which causes more pronounced increase in p-type conductivity in the irradiated p-a-Si film compared to the irradiated n-a-Si.

The conductivity anisotropy observed in all femtosecond laser-irradiated samples can be explained by form anisotropy of the LIPSS [3]. As seen in Table 3, the conductivity along the LIPSS is higher than in the direction orthogonal to them. This effect is more prominent in the case of n-a-Si and is consistent with the formation of a relief with much greater relative depth, comparable to the overall film thickness, which can be seen in Figure 5e,f.

The anisotropy form of the LIPSS can affect the electrical conductivity as follows. The LIPSS produced by femtosecond laser radiation on the surface of all samples represent a one-dimensional grating, consisting of ridges and valleys ablated between them, with a depth of up to ~350 nm. Thus, when the external electric field ***E*** is directed along the LIPSS the transport of charge carriers occurs along the ridges of the grating, while in the case when ***E*** is orthogonal to the LIPSS, the charge carriers encounter the valleys of the grating, where the film is much thinner or, in case of sample N2, is completely absent. In addition, such one-dimensional high-contrast relief can have a strong depolarizing effect on an external electric field when it is applied perpendicularly to LIPSS ridges [3].

However, we should note that in the case of samples P1 and P2, the formed relief is weakly ordered and relatively shallow (from 100 to 300 nm) compared to the thickness of the p-a-Si film itself (~1.25 μm). Despite that, the electrical anisotropy of modified p-a-Si is quite large: the conductivity of the P1–1 and P2–1 area (***E*** ∥ LIPSS) is from 3.5-to-5 times higher than P1–2 and P2–2 (***E*** ⊥ LIPSS). In this case, the high conductivity difference in mutually orthogonal directions may be additionally influenced by the shape anisotropy of silicon nanocrystals and their preferential orientation during formation under the action of high-power polarized laser radiation in the bulk of the film. Previously, the anisotropic silicon nanocrystals formation by polarized femtosecond laser pulses for an undoped a-Si film was shown in our work [3], where preferential orientation of the nanocrystals was proposed based on an analysis of the Raman spectra of irradiated films. In addition, the possibility of elongated ellipsoidal nanocrystal formation in the high-power polarized laser radiation field was theoretically predicted in the work [52].

The observed CVC nonlinearity (Figure 7b) may be explained by the presence of nonuniformly distributed nc-Si phase within the bulk of the films. The conduction occurs in both amorphous and nc-Si phases of the film, while the band gaps of these phases are known to differ (1.12 eV for crystalline silicon and ~1.8 eV for a-Si), and the band edges do not coincide. Therefore, the potential barriers, presumably, can appear for the transport of charge carriers between the amorphous and crystalline phases. Such barriers may exist for in-plane current, where they may be caused by uneven *f_C_* distribution within LIPSS [18], as well as the above-mentioned anisotropy of the silicon nanocrystals. Additionally, nonlinear conductivity of the irradiated films may be associated with the Schottky barrier formation in electric contacts at the metal/semiconductor interface. The absence of such nonlinearity in the initial films, as well as irradiated n-a-Si samples, could be attributed to their low conductivity, resulting into “quasiomic” CVC behavior. Conversely, for the p-a-Si films, where the conductivity increased by 5–6 orders after femtosecond laser irradiation, the Schottky barrier may significantly impact the CVC.

## 5. Conclusions

We have demonstrated the formation of LIPSS by femtosecond laser pulses on the surface of p-a-Si and n-a-Si films. The formed surface relief is one-dimensional and directed orthogonally to the polarization of the modifying radiation, while the depth and period of the relief depends on the number of acting laser pulses, *N*. The LIPSS are classified as LSFL, in which their period decreases while their depth increases with higher *N*, due to a shift in the surface plasmon-polariton resonant wavelength.

Variation of the Raman scattering spectra caused by the laser irradiation evidences that surface structuring is accompanied by simultaneous laser-induced nanocrystallization of the films. The nanocrystalline silicon phase volume fraction *f_C_* in all cases is inhomogeneous within the film depth. The value of *f_C_* decreased with depth from ~80% to ~40% and from ~20% to ~10%—in p-a-Si and n-a-Si-films, respectively—which is explained by the higher laser radiation absorption in the upper layers of the film.

The conductivity of all modified films increased after laser irradiation. For p-a-Si the increase was up to 6 orders of magnitude, from ~10^−9^ S/cm to ~10^−2^ S/cm, due to the formation of a highly conductive crystalline phase, whereas in n-a-Si films the conductivity increased only up to 2 orders of magnitude, which is associated with a lower *f_C_* (<20%), as well as more intense ablation and higher doping efficiency in the initial film.

The conductivity in the irradiated films is up to 1 order of magnitude higher along the LIPSS ridges. Observed conductivity anisotropy may be explained by the form anisotropy of both LIPSS and silicon nanocrystals formed, by the femtosecond laser treatment. The LIPSS strongly affect the anisotropy of conductivity in case of a deep surface relief comparable to the film thickness. Besides, in case of shallow and weakly ordered LIPSS the preferential orientation of the elongated silicon nanocrystals may additionally influence such anisotropy. The observed CVC nonlinearity may be attributed to uneven *f_C_* distribution, presumably causing potential barriers for the in-plane carriers transport, as well as to formation of the Schottky barriers at the metal/semiconductor interfaces.

The obtained results demonstrate potential for designing new electrically anisotropic planar elements of photovoltaics and optoelectronics by femtosecond DLW.

## Figures and Tables

**Table 1:** Sample preparation parameters and formed LIPSS periods.

Sample	Doping Type	Thickness (μm)	Fluence (J/cm^2^)	Scanning Speed *V* (μm/s)	Laser Pulses Number *N*	LIPSS Period (μm)
P1	p-a-Si	1.25 ± 0.03	0.3 ± 0.1	200	30	1.1 ± 0.1
P2				50	120	0.84 ± 0.07
N1	n-a-Si	0.35 ± 0.04	0.15 ± 0.05	200	30	1.06 ± 0.03
N2				50	120	0.98 ± 0.01

**Table 2:** Silicon nanocrystals size and the nc-Si phase volume fraction within the modified samples.

Sample	Nanocrystal Size *d_nc-Si_* (nm)		Nc-Si Phase Volume Fraction *f_C_* (%)	
	Front	Back	Front	Back
P1	5 ± 2	1.0 ± 0.5	79 ± 13	41 ± 6
P2	5 ± 2	1.0 ± 0.5	84 ± 7	42 ± 5
N1	3 ± 1	3 ± 1	18 ± 3	7 ± 3
N2	4 ± 1	3 ± 1	19 ± 2	11 ± 5

**Table 3:** Conductivity of the initial and modified p-a-Si and n-a-Si samples.

Sample	*E* ∥ LIPSS		*E* ⊥ LIPSS	
	Area	Dark Conductivity (S/cm)	Area	Dark Conductivity (S/cm)
P1	P1–1	(12.5 ± 0.6) × 10^−3^	P1–2	(3.5 ± 0.9) × 10^−3^
P2	P2–1	(11 ± 5) × 10^−3^	P2–2	(2.1 ± 1.3) × 10^−3^
Unmodified p-a-Si		(2.2 ± 0.5) × 10^−9^		
N1	N1–1	(11 ± 5) × 10^−5^	N1–2	(8.3 ± 0.4) × 10^−6^
N2	N2–1	(8.1 ± 0.7) × 10^−6^	N2–2	(8.6 ± 0.1) × 10^−7^
Unmodified n-a-Si		(1.9 ± 0.5) × 10^−6^		

## Data Availability

Not applicable.

## Supplementary Materials

The following supporting information can be downloaded at: https://www.mdpi.com/article/10.3390/ma15217612/s1, Supplementary file S1: Calculation of the energy absorbed by p-a-Si and n-a-Si films under the action of a single femtosecond laser pulse; Supplementary file S2: X-ray diffraction analysis of the initial and modified p-a-Si and n-a-Si films. References [36,37] are cited in the supplementary materials.
