# Supplementary material for: Femtosecond Laser Fabrication of Anisotropic Structures in Phosphorus- and Boron-Doped Amorphous Silicon Films
Source: Materials (Basel). 2022 Oct 29;15(21):7612. doi: 10.3390/ma15217612 (PMC9654927; doi:10.3390/ma15217612)
Supplement: Supplementary file 1 [file materials-15-07612-s001.zip › materials-1960332-supplementary.pdf]

Supplementary file S1: Calculation of the energy absorbed by p-a-Si and n-a-Si films under the action of a single femtosecond laser pulse

An a-Si film with greater thickness requires a greater amount of energy per pulse to achieve crystallization of its volume, since the volume of the film affected by the laser pulse is larger. In order to compare the results of different films irradiation, we calculated the energy absorbed by films with different thicknesses (350-nm-thick n-a-Si film and 1250-nm-thick p-a-Si) under the action of single laser pulses with the corresponding fluences.

The calculations were as follows. First, we determined the intensity of the radiation I_n and I_p transmitted through n-a-Si and p-a-Si film respectively. We should note, that at the radiation wavelength λ=1250 nm used in our experiments the absorption coefficient α of the a-Si is small (α=10 cm^{-1} according to Ref. [36]), while the laser radiation intensity was on the order of 10^{12} W/cm^2. At such high radiation intensity two-photon absorption should considerably contribute to the a-Si film absorption. Therefore, we used the following differential equation for transmitted intensity I with depth z:

$$\mathrm{d}I/\mathrm{d}z = -\,\alpha I - \beta I^2, \quad \text{(S1)}$$

where β=37 cm/GW [37] is the two-photon absorption coefficient for a-Si. The equation (S1) was solved using Wolfram Mathematica software, for the initial radiation intensity $I_{0n} = 1\cdot10^{12}$ W/cm^{2}and $I_{0p} = 2\cdot10^{12}$ W/cm^2. The initial radiation intensity was estimated as:

$$I_{0n} = F_n/\tau,\ I_{0p} = F_p/\tau, \quad \text{(S2)}$$

where $\tau = 150$ fs is the pulse duration, F_n=0.15 J/cm^2 and F_p=0.3 J/cm^2 are the laser fluences for n-a-Si and p-a-Si, respectively. The obtained from (S1) transmitted intensity values are $I_n = 4.3\cdot10^{11}$ W/cm^2 and $I_p = 1.9\cdot10^{12}$ W/cm^2 for n-a-Si and p-a-Si films, respectively. Hence we estimated the value of the energy E_n and E_p absorbed by n-a-Si and p-a-Si films, respectively, when exposed to a single pulse:– –

$$E_n = (I_{0n}-I_n)\tau S,\ E_p = (I_{0p}-I_p)\tau S, \quad \text{(S3)}$$

where $S \approx 0.07$ mm^2 is the laser spot square area. According to equations (S3) the energy absorbed from a single laser pulse by n-a-Si film $E_n \approx 150$ nJ and by p-a-Si film $E_p \approx 480$ nJ. Thus, the energy absorbed by the films per pulse differs by a factor of ~3.2, which is comparable to the difference in the volume affected by laser pulse for these films (3.6 times).

Supplementary file S2: X-ray diffraction analysis of the initial and modified p-a-Si and n-a-Si films

X-ray diffraction (XRD) experiments were carried out on a PANalytical Empyrean, a multi-purpose X-ray diffractometer with a vertical goniometer. An X-ray tube with a copper anode (λ = 1.5418 Å) was used as an X-ray source. The XRD patterns were taken for (2θ) diffraction angle ranging from 5° to 100° for 50 minutes. Processing of diffraction patterns and phase analysis of the samples were carried out using the HighScore Plus software.

The obtained XRD patterns for both the initial sample (Figure S1a) demonstrate wide band at the diffraction angle at around 23° corresponding to (111) orientation of SiO_2, which is explained by presence of a thick (about 1 mm) quartz glass substrate, as well as narrow lines at the diffraction angles around 38°, 45°, 65° and 78° corresponding to (111), (002), (022), and (113) facets of Al and Ag, present in the electrical contacts. In this case, there are no lines corresponding to crystalline silicon in the diffraction pattern, which indicates the amorphous structure of the film. Features corresponding to amorphous silicon are also could not be observed, since the signals corresponding to the amorphous Si phase were too weak from a film with a thickness of 350 to 1250 nm. In the diffraction pattern for the irradiated sample (Figure S1b), in addition to the above-mentioned lines, there were observed lines at the diffraction angles around 28°, 48° and 56°, corresponding to (111), (022) and (113) facets of Si fcc cubic lattice, which indicates the crystallization of the film after exposure to femtosecond laser pulses.

Nevertheless, oxidation of the amorphous silicon film as a result of femtosecond laser irradiation cannot be examined based on the performed analysis mainly due to presence of the quartz glass substrate.

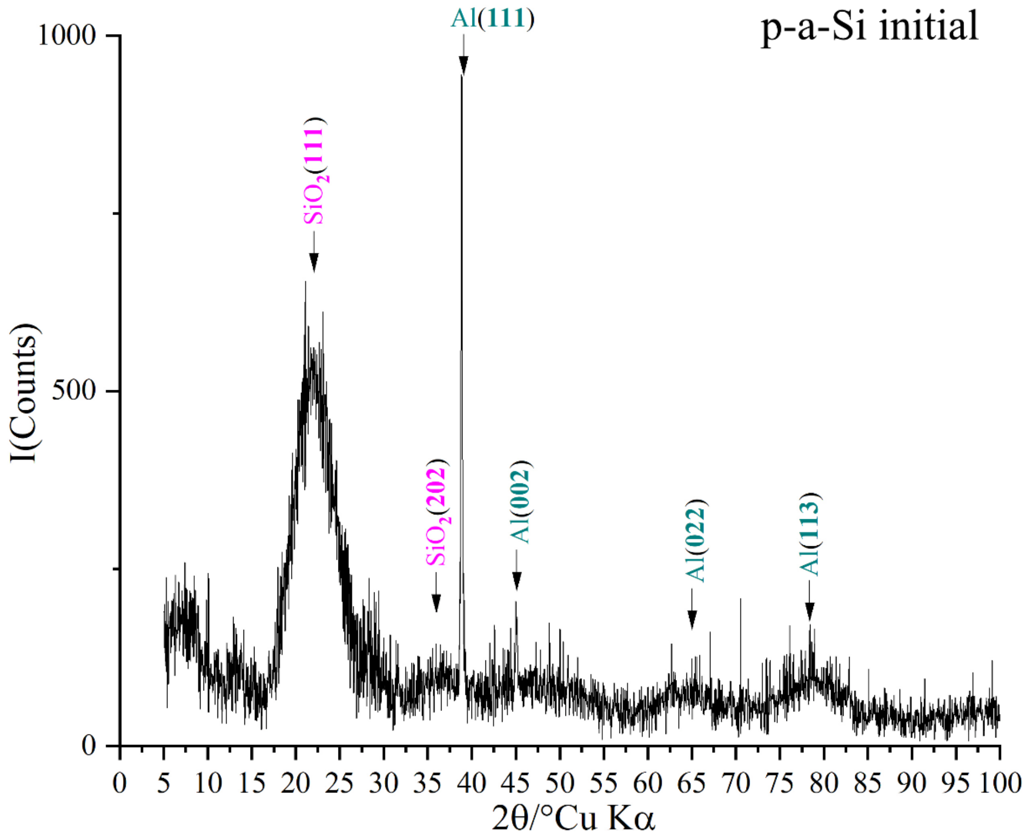

(a)

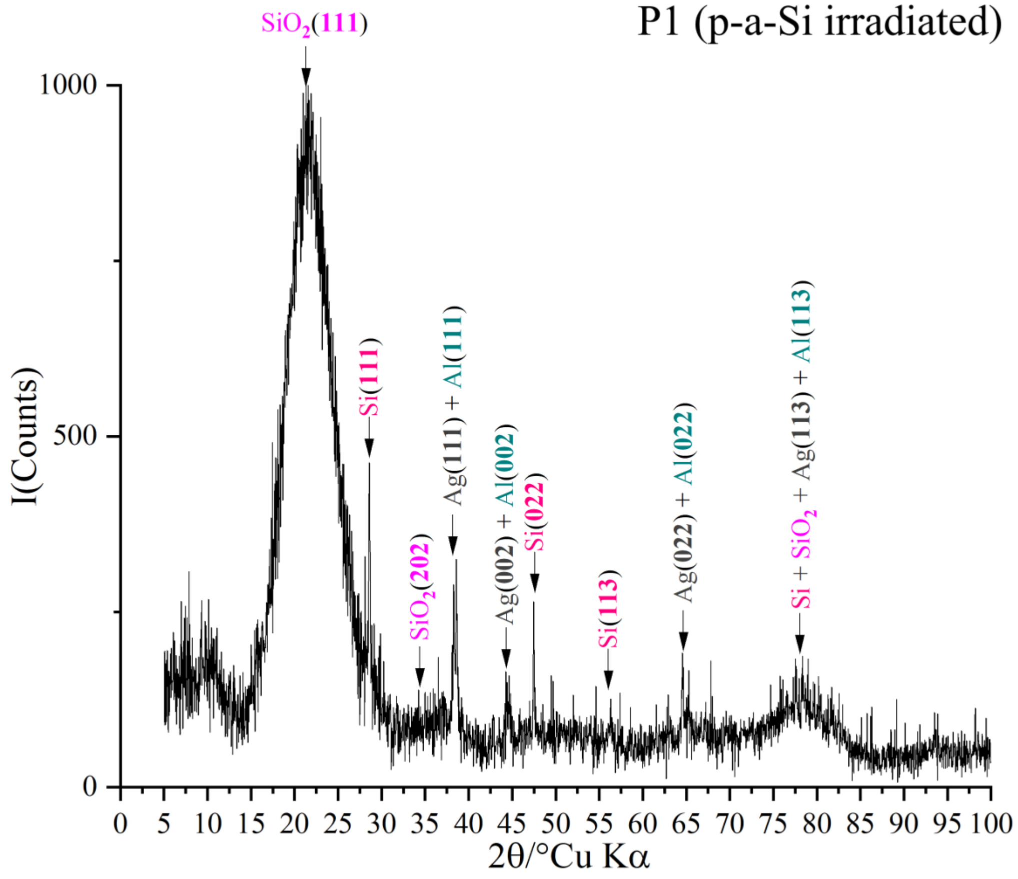

(b)

Figure S1. XRD patterns of the (**a**) initial p-a-Si film, and (**b**) irradiated sample P1
